# Health policy and systems research publications in Latin America warrant the launching of a new specialised regional journal

**DOI:** 10.1186/s12961-020-00565-1

**Published:** 2020-06-05

**Authors:** Miguel Angel González Block, Juan Arroyo Laguna, Oscar Cetrángolo, Pedro Crocco Ábalos, Ramiro Guerrero, Daniela Riva Knauth, Abdul Ghaffar, Patricia Pavón León, María del Rocío Saénz, Rosanna González McQuire, Beatriz Martínez Zavala, Emilio Gutiérrez Calderón

**Affiliations:** 1 Universidad Anáhuac, Instituto Nacional de Salud Pública, Mexico City, Mexico; 2 Facultad de Ciencias Sociales, Escuela de Gobierno y Políticas Públicas, Pontificia Universidad Católica del Perú, Lima, Peru; 3 Facultad de Ciencias Económicas de la UBA, Buenos Aires & Instituto Interdisciplinario de Economía Política, Buenos Aires, Argentina; 4 Escuela de Salud Pública, Universidad de Chile, Santiago, Chile; 5 Centro de Estudios en Protección Social y Economía de la Salud, Universidad ICESI, Cali, Colombia; 6 Departamento de Medicina Social, Universidade Federal do Rio Grande do Sul, Porto Alegre, Brazil; 7 Alliance for Health Policy and Systems Research, World Health Organization, Geneva, Switzerland; 8 Instituto de Ciencias de la Salud, Universidad Veracruzana, Xalapa, Veracruz Mexico; 9 Universidad de Costa Rica, San Jose, Costa Rica; 10 Evisys Consulting, Mexico City, Mexico; 11 Universidad Nacional Autónoma de México, Mexico City, Mexico

**Keywords:** Health policy and systems research, Health research capacity strengthening, Scientometrics, Latin America

## Abstract

**Background:**

Scientific journals play a critical role in research validation and dissemination and are increasingly vocal about the identification of research priorities and the targeting of research results to key audiences. No new journals specialising in health policy and systems research (HPSR) and focusing in the developing world or in a specific developing world region have been established since the early 1980s. This paper compares the growth of publications on HPSR across Latin America and the world and explores the potential, feasibility and challenges of innovative publication strategies.

**Methods:**

A bibliometric analysis was undertaken using HPSR MeSH terms with journals indexed in Medline. A survey was undertaken among 2500 authors publishing on HPSR in Latin America (LA) through an online survey, with a 13.1% response rate. Aggregate indicators were constructed and validated, and two-way ANOVA tests were performed on key variables.

**Results:**

HPSR publications on LA observed an average annual growth of 27.5% from the years 2000 to 2018, as against 11.4% worldwide and yet a lag on papers published per capita. A total of 48 journals with an Impact Factor publish HPSR on LA, of which 5 non-specialised journals are published in the region and are ranked in the bottom quintile of Impact Factor. While the majority of HPSR papers worldwide is published in specialised HPSR journals, in LA this is the minority. Very few researchers from LA sit in the Editorial Board of international journals. Researchers highly support strengthening quality HPSR publications through publishing in open access, on-line journals with a focus on the LA region and with peer reviewers specialized on the region. Researchers would support a new open access journal specializing in the LA region and in HPSR, publishing in English. Open access up-front costs and disincentives while waiting for an Impact Factor can be overcome.

**Conclusion:**

Researchers publishing on HPSR in LA widely support the launching of a new specialised journal for the region with a vigorous editorial policy focusing on regional and country priorities. Strategies should be in place to support English-language publishing and to develop a community of practice around the publication process. In the first years, special issues should be promoted through a priority-setting process to attract prominent authors, develop the audience and attain an Impact Factor.

## Introduction

Health policy and systems research (HPSR) is a relatively new field of specialisation in which diverse health and social science disciplines are converging with the aim of producing knowledge on the organised societal response to population health needs [1]. HPSR as a specialised field was initiated in advanced industrialised countries where increasing health expenditure associated to technological development and the epidemiological transition posed efficiency and equity challenges. Research became closely associated to policy and managerial reforms, giving rise to a movement towards evidence-based decision-making. Thus, research capacity and journals specialised in HPSR arose in high-income countries to focus research on national and regional priorities.

HPSR for low- and middle-income countries was first prioritised by high-income country development assistance agencies and academic institutions as well as by multilateral organisms through key initiatives such as the Council for Health Research for Development, the WHO-led Ad Hoc Committee on Health Research Relating to Future Intervention Options, the Tropical Disease Research and Training Programme, and the Alliance for Health Policy and Systems Research. More recently, the Millennium Development Goals and the movement towards Universal Health Coverage led to further strengthening of research capacity [2, 3].

In the early 2000s, the Alliance for Health Policy and Systems Research reported the existence of a diversified research agenda in developing countries, focusing on health system-wide problems as well as on health services research. Greater attention was given to the community and hospital levels, with less emphasis on primary care or on topics such as policy process and information systems. Upper middle-income countries, including most in Latin America (LA), had a greater inclusion of topics such as health insurance, decentralisation, local health systems, equity and the policy process, suggesting the importance that researchers of the region give to the study of prevalent problems rooted in health system segmentation and inequity [4]. The growth in HPSR publications, including authors from low- and middle-income countries, surpasses growth of HPSR papers in general, and even of life and biomedical sciences in general, thus attesting to the growth in capacity associated to the above-mentioned initiatives, among others [5].

HPSR capacity in developing countries has been strengthened across a wide range of health research system components, including training, the utilisation of research for policy-making and programme management [6], and research funding [7]. However, no efforts have been made to address the need for research journals specialised in HPSR for developing countries, with the salient exception of *Health Policy and Planning*, launched by the London School of Hygiene and Tropical Medicine in 1986, the sole HPSR-specialised journal focusing in the developing world. While it is recognised that the future of HPSR will depend on diversifying its agenda, on enhancing the collaboration across health system actors and on its embedding in policy processes [8], attention should also be given to research publication as a central component of the HPSR system.

Scientific journals play a critical role in research result recording, validation and dissemination, while their limitations for policy-making have been recognised and addressed through research translation [9], implementation research [10], and research synthesis and translation platforms [11]. Journals are increasingly vocal for the identification of research priorities and the targeting of research results to key audiences [12]. Furthermore, journals are playing a key role in the development of communities of practice through special issues and commissions [13]. The number of scientific articles being published [14], their medium of publication [15], and the reading public are growing and diversifying [16]. Journals’ specialisation has increased in thematic and geographic scope, partly as a result of a growing trend in specialisation across all scientific disciplines [17]. Journal specialisation has been boosted by the rise of on-line, open access journals, whose number has grown faster than subscription journals, attaining a similar citation impact as subscription journals [18].

Research journals, particularly those on public health and HPSR, are increasingly playing an active role to promote research capacity and, especially, to influence policy. Given that HPSR is a highly contextualised field of study, it can be questioned if journals dedicated to publishing mostly on high-income country issues can also promote developing country priorities or peer review papers to effectively promote research capacity strengthening and policy impact. Arguably, academic pressure to publish on international journals with an impact factor (IF) is a powerful incentive to submit to international journals regardless of the specific benefits received. This situation also creates barriers to the emergence of specialised journals more appropriate to specific regions and topics.

The Latin American Network of Health Policy and Systems Research was established in 2012 to strengthen research capacity in the region through supporting young researchers and discussing the research agenda and capacity strengthening needs. This paper aims to address the Network’s remit through exploring two related research questions, namely whether researchers are producing quality publications on HPSR for the region at rates comparable to those observed for the world as a whole and which are the alternative publication strategies to enhance the potential for quality HPSR publications, analysing their feasibility and challenges.

## Methods

To address the production of quality HPSR publications in the LA region, the relative growth of HPSR journal articles and their concentration within journals with an IF were analysed. Articles of any type published on HPSR globally and for LA and indexed in Medline between 2000 and 2018 were selected using relevant MeSH terms (Box 1). The countries of the institutions to which each author was affiliated were classified as high income or as middle/low income. Chile was classified as middle income, even though it became a high-income country in 2013. The LA region includes all Spanish- and Portuguese-speaking countries in the continent and in the Caribbean. The analysis of HPSR articles by journal focused on IF Journals publishing the highest number of HPSR articles between 2011 and 2016. Articles published in mixed-scope journals were classified using the same subject and country MeSH terms, while all articles published by HPSR specialised journals were quantified according to country focus. IF journal board membership was analysed through journal websites to determine the percentage of LA participants based on the identification of Spanish or Portuguese surnames. To this end, the list of journal board members published in each journal’s website was inspected and a headcount was undertaken. IFs for 2016 were identified for each journal through Scimago Journal & Country Rank (https://www.scimagojr.com/journalrank.php) and confirmed in the journal’s webpage. Journals were arranged into IF quintiles excluding outliers (*PLoS One* and *Lancet*). Emails of all authors were extracted for the period of 2011 to 2017 and used for the survey. Data was downloaded to Excel for processing. World and LA population totals were used to estimate the share of papers produced, based on estimates by Worldometers for current populations (https://www.worldometers.info/world-population/caribbean-population/).

A survey was undertaken to address the question of publication strategies favoured by authors of HPSR on LA. A total of 2501 authors were identified from the Medline citations retrieved for the previous analysis. Out of this total, 2287 had valid emails. Authors were invited to answer a questionnaire in Spanish or English through Survey Monkey^©^ between May 3 and June 7, 2019. The survey included 10 batteries of questions in 4 sections – characteristics and country of residence of respondents; potential of a new specialised journal focusing on HPSR in LA, edited with the support of regional resources and guidance (Table 3); feasibility of an open access business model (Table 4); and challenges in waiting a number of years prior to attaining an IF (Table 5). Measurement scales for all batteries were of the Likert type. Responses were downloaded in XLS format and processed in SPSS Statistics Version 2.3.

The respondent age, percentage of time dedicated to research and number of articles published in peer-reviewed journals were categorised for analysis. Country of residence was categorised into two regions, namely LA and the United States, Canada or Europe. Six authors from outside these regions were excluded for regional analysis (Additional file 1: Appendix 2). Aggregate indicators for ‘Potential’, ‘Feasibility’ and ‘Challenges’ of publication strategies were constructed by adding individual responses. The indicator of ‘Potential’ excluded responses to preferences for journal specialisation, as these responses were analysed as independent variables. Responses for ‘Feasibility’ were inverted as required to point in the same positive direction. The internal consistency of indicators was validated using Cronbach’s Alpha. Descriptive statistics were performed for all the variables and two-way ANOVA tests were performed to identify the association between individual characteristics and preferences for editorial strategies with respect to ‘Potential’, ‘Feasibility’ and ‘Challenges’.

No ethics approval was deemed necessary for this investigation as no human subjects were placed at risk. Researchers participating in the consultation were informed that their responses would be confidential.

Box 1. MeSH keywords Health Policy and Systems Research

“health manpower” OR “health personnel” OR “health promotion” OR “health policy” OR “health services research” OR “health services” OR “health care economics” OR “healthcare organizations” OR “healthcare economics” OR “healthcare organisations” OR “health care organizations” OR “health care organizations” OR “health services administration” OR “healthcare quality access evaluation” OR “health care quality access evaluation”.

Latin American countries or region

“Brazil” OR “Mexico” OR “Argentina” OR “Colombia” OR “Peru” OR “Chile” OR “Venezuela” OR “Ecuador” OR “Dominican Republic” OR “Guatemala” OR “Panama” OR “Costa Rica” OR “Bolivia” OR “Uruguay” OR “Paraguay” OR “El Salvador” OR “Honduras” OR “Nicaragua” OR “Cuba” OR “Puerto Rico” OR “Latin America” OR “South America” OR “Central America” NOT “New Mexico”.

Journal search

“Lancet” OR “PLoS Med” OR “Lancet Global Health” OR “Bull World Health Organ” OR “Health Aff” OR “Am J Public Health” OR “Implementation Sci” OR “Health Policy Plan” OR “Value in Health” OR “PLoS One” OR “Int J Pub Health” OR “Arch Med Research” OR “J Health Econ” OR “J Pub Health” OR “BMC Public Health” OR “Health Econ” OR “Health Serv Res” OR “J Pub Health Policy” OR “Hum Resour Health” OR “J Health Serv Res Policy” OR “Int J Equity Health” OR “Health Policy” OR “BMC Health Serv Res” OR “Health Econ Policy Law” OR “Glob Pub Health” OR “Public Health” OR “Persp Pub Health” OR “J Health Polit Policy Law” OR “Ann Glob Health” OR “J Prim Health Care” OR “Revista de Saude Publica” OR “Int Health” OR “Salud Pública de México” OR “Cadernos de Saude Publica” OR “Int J Health Plan Manage” OR “Int J Health Serv” OR “PanAmerican J Pub Health” OR “Inquiry” OR “J Health Organ Manage” OR “Gaceta Medica de Mexico”.

## Results

### Bibliometric analysis

**Fig. 1:**
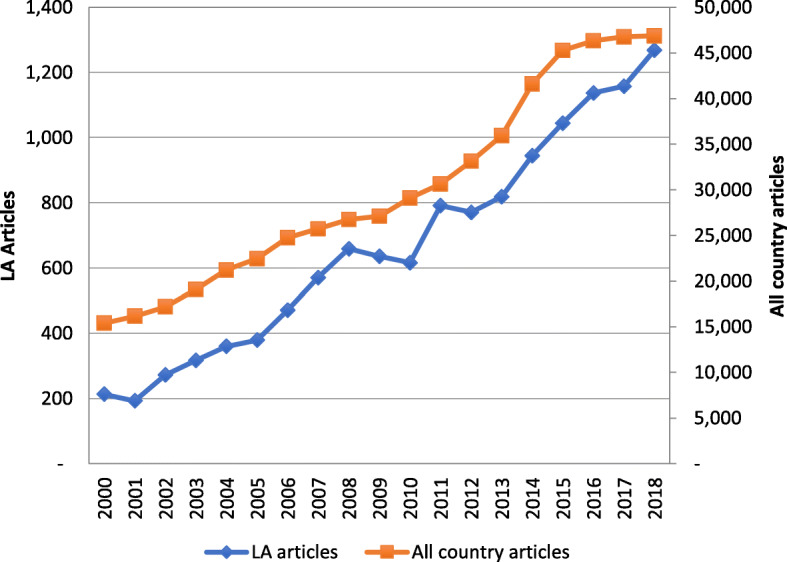
Articles on health policy and systems research with a focus on Latin American countries. Total Medline citations 2000–2017

A total of 213 articles were published on HPSR on LA in 2000 and increased by a factor of 5.4 for a total of 1268 for 2018, an average annual growth of 27.5% (Fig. 1). Globally, a total of 15,393 articles were published in 2000 and increased to 46,853 for 2018. The growth factor within this period was of 3.0 for total papers and of 6.0 for LA articles. The trend both regionally and globally seems to have been affected by the 2009 global recession but recovered its pace since 2013. While the LA region contributed with 1.4% of total articles for 2000, for 2018, its contribution doubled to 2.7%. This attests to a higher rate of growth for the LA region at 27.5% per annum on average, as against 11.4% globally. Total papers for LA were 1.95 per million inhabitants as against 6.03 for the world as a whole.

Between 2000 and 2014, high-income country authors participated in at least 27% and up to 36% of total yearly papers. Out of the total number of papers, 26% (222 on average per year) were published in journals with an IF.

A total of 48 journals with an IF were identified as publishing in the field of HPSR in 2016. Of these, 20 were fully specialised in the topic and the rest published articles within a broader medical and public health context. Five of these journals were published with a LA or pan American remit and by institutions in the region, of which three ranked at the bottom IF quintile distribution (IF = 0.27–0.98) and two in the fourth quintile (IF = 1.0–1.9). Specialised journals published 56.6% of the total papers on HPSR and 18.4% of HPSR papers on LA. The information available on the websites of 31 IF journals suggests that, among those specialised in HPSR, Editorial Board members from the LA region account for 1.5% of the total. This same figure is 2.8% for mixed-scope journals published outside the region and 41% for mixed-scope journals published within the region.

The top 10 journals publishing HPSR papers on LA accounted for 79% of articles published between 2011 and March 2016, each publishing 140 papers per year on average (Table 1). The top 4 journals are edited in LA, all of mixed scope and publishing 65% of regional HPSR papers. The remaining 6 journals – of which only 2 specialise in HPSR – publish at most 8.5% of regional HPSR papers, while up to 4.2% of their Editorial Board members are from LA. A total of 2501 individual HPSR article authors were identified between 2011 and March 2019. Considering the period up to March 2016, 13.5% of authors (*n* = 102) from 90 institutions accounted for 50.4% of papers.

**Table 1:** Articles on health policy and systems research (HPSR) in Latin America (LA) published by top 10 Impact Factor (IF) journals, 2011–2016

	Total number of articles published on all topics	Articles on HPSR	Percentage of articles on HPSR	HPSR articles on LA	Percentage of HPSR articles on LA	Percentage of Editorial Board members from LA	IF (2016)
Cadernos de Saude Publica	1451	352	24.3	245	69.6	NA	0.98
Revista de Saude Publica	707	173	24.5	120	69.4	97.1	1.21
PanAmerican Journal of Public Health	697	180	25.8	119	66.1	58.8	0.76
Salud Pública de México	620	131	21.1	92	70.2	61.5	1.03
PLoS One	135,988	2135	1.6	87	4.1	1.5	3.2
BMC Public Health	6424	1916	29.8	54	2.8	1.9	2.26
Health Policy and Planning	632	632	100.0	54	8.5	0.0	3.47
Lancet	8531	1251	14.7	46	3.7	4.2	45.2
BMC Health Services Research	2781	2781	100.0	37	1.3	0.9	1.17
Value in Health	3628	262	7.2	27	10.3	0.0	3.28
Total	161,459	9813	6.1	881	9.0		

#### Researcher survey

Out of the 2287 authors contacted by email, 299 responded (13.1%) (Table 2). Respondents declared having published, on average, 39 peer-reviewed research papers in their careers, with a median of 21. Up to 35.3% of respondents are fully dedicated to research, which together with age and publications, suggest a high level of experience in HPSR. National government research funding institutions are the most important source according to 42.1% of respondents, with other sources such as own institutions, non-governmental organisations and international sources accounting for the remainder.

**Table 2:** Individual author characteristics

Variable	*N*	Characteristics	*n*	%
Sex	292	Female	158	54.1%
		Male	134	45.9%
Age, years	298	< 40	82	27.5%
		40–50	96	32.2%
		> 50	120	40.3%
Main funding source	299	National	126	42.1%
		Other	173	57.9%
Time allocated to research, % of working hours	271	< 25	43	16%
		25–50	68	25%
		50–75	97	36%
		75–100	63	23%
Papers published in peer-reviewed journals	266	< 21	130	48.9%
		> 21	136	51.1%
Specialisation in health policy and systems research	272	Complete	96	35.3%
		Partial	176	65.7%
Region of residence	291	Latin America	233	80.1%
		United States, Canada, Europe	58	19.9%

Respondents declared 282 institutions of affiliation in 28 countries, with Brazil being the most frequent (32.8% of the total), followed by Mexico and the United States (Additional file 1: Appendices 1 and 2). Respondents from the United States, Canada and Europe represented 19.9% of the total, suggesting a moderate level of interest on LA by researchers from outside the region.

The indicator of ‘Potential’ of the editorial strategy had a Cronbach Alpha of 0.71, within the 0.7–1.0 range and therefore acceptable. The Cronbach Alpha for ‘Feasibility’ and ‘Challenges’ were 0.662 and 0.667, respectively, considered as on the limits of acceptability. ‘Potential’ was the only indicator with significant associations in the two-way ANOVA tests with respect to HPSR and regional specialisation.

##### Potential of the editorial strategy

Respondents prioritised an editorial policy emphasising open access for all papers, with up to 70.9% responding that this would greatly facilitate dissemination to key audiences (Table 3). Commitment to publish within 3 months and a robust online submission platform followed, with 65% of respondents in both cases considering this would greatly facilitate publication. Of less importance, although still considered important facilitators, were policies to ensure the majority of editorial board members are prominent LA researchers and policy-makers as well as publication by a well renowned, international publisher and English language publication with optional Spanish or Portuguese translations. Specialisation of a journal in HPSR was considered as a facilitator of quality publications by 94% of respondents, with 49.1% considering this a very important facilitator. Specialisation of a journal in the LA region was considered as a facilitator of quality publications by 86% of respondents, of whom 47.2% considered it a very important facilitator.

**Table 3:** Potential of a new specialised journal on health policy and systems research (HPSR) in Latin America – To what extent do you consider the following factors as facilitators or obstructers for the publication of quality HPSR articles in Latin America?

	n	Greatly facilitate	Facilitate	Neither facilitate nor obstruct	Obstruct	Greatly obstructs
The journal has an open access policy for all articles, enabling free dissemination to key audiences	265	70.9%	23.4%	5.3%	0.4%	0.0%
The publisher commits to a 3-month publication period, from submission to publication	265	65.3%	29.4%	3.8%	0.8%	0.8%
The journal publishes on-line using a comprehensive, robust platform	266	65.0%	32.7%	2.3%	0.0%	0.0%
The journal has a rigorous peer-review process striving to considerably involve recognised health systems researchers from Latin America	265	59.2%	33.6%	5.3%	1.8%	0.8%
The journal promotes special issues on highly relevant subjects for Latin American health systems	266	58%	36%	5%	1.1%	0.8%
The majority of the journal’s Editorial Board members are prominent researchers and policy-makers from Latin America	266	43.2%	49.8%	13.9%	2.6%	0.4%
The journal is published by a reputable, internationally recognised firm	266	47.4%	36.8%	13.9%	1.1%	0.8%
Papers are published in English, with optional Spanish or Portuguese translations	266	46.6%	31.2%	10.5%	9.4%	2.3%
The journal specialises on the Latin American region	267	47.2%	39.3%	10.1%	3.4%	0.0%
The journal specialises on HPSR	267	49.1%	43.1%	6.4%	1.5%	0.0%

The indicator of ‘Potential’ of the proposed editorial policy was positively associated with an editorial strategy of specialisation in HPSR (*P* = 0.012) and particularly with specialisation in the LA region (*P* = 0.0003).

##### Feasibility and challenges of open access publications

Open access publications are preferred, with up to 76% of respondents supporting them or highly supporting them (Table 4). Up to 52% of authors agreed that their institutions encourage them to publish in open access publications. However, only 29% of respondents agreed that payment for open access is not a publication barrier, with 32% responding that they highly disagree with payment not being a barrier. Only 15% agreed or highly agreed that their institutions have agreements with open access publishers, while 15% were not sure and responded that they ‘neither agree nor disagree’ with this statement.

**Table 4:** Feasibility of a new research journal on health policy and systems research in Latin America – To what extent are you in agreement or disagreement with the following statements regarding open access publications? (*n* = 267)

	Highly agree	Agree	Neither agree nor disagree	Disagree	Highly disagree
I prefer to publish in open access journals	48.3%	27.7%	17.6%	4.9%	1.5%
My institution encourages me to publish in open access journals	25.1%	27.0%	33.0%	9.7%	5.2%
Paying for open access journals does not represent a major barrier for my publications	7.5%	13.5%	10.9%	36.3%	31.8%
I can easily ear-mark project funds to pay for my publications in open access journals	3.4%	14.6%	9.0%	39.0%	34.1%
My academic institution has an agreement to pay for my publications with an open access publisher	4.5%	10.9%	14.6%	27.0%	43.1%

Respondents showed a mixed opinion regarding challenges in publishing in journals without an IF (JWIF) (Table 5). Up to 32% declared a degree of agreement that they cannot allocate research funding to publish in JWIF, while 50.4% agreed that they have to commit to publishing in journals with an IF in their research proposals. However, up to 64% of respondents agreed that they would consider publishing in JWIF if research results were directed to key audiences, while 74.5% would do so as part of special issues along with distinguished authors. Up to 47.4% of respondents agreed that they would be willing to publish in JWIF if they did not have to pay to do so.

**Table 5:** Challenges to publishing a new journal awaiting an Impact Factor – To what extent do you agree or disagree with the following statements regarding the Impact Factor of a journal?

	n	Highly agree	Agree	Neither agree nor disagree	Disagree	Highly disagree
I am forbidden to use grant money to publish main results in journals without an Impact Factor^a^	266	12.4%	20.3%	29.3%	19.5%	18.4%
I state in my grant proposals that results will be sent for publication to high Impact Factor journals^a^	266	20.3%	30.1%	27.4%	12.8%	9.4%
I would publish in a journal without an Impact Factor if it targets results to key audiences	267	23.6%	40.4%	17.6%	11.2%	7.1%
I would publish my results in a journal without an Impact Factor if my paper was part of a special issue with the participation of prominent authors	267	29.6%	44.9%	6.4%	12.0%	7.1%
I would publish in a journal without an Impact Factor if I did not have to pay for its publication	266	18.8%	28.6%	21.4%	17.3%	13.9%

^a^ Agreement represents a barrier to the launch of a journal without an Impact Factor

The aggregate indicators of ‘Feasibility’ and ‘Challenges’ of an editorial strategy including open access publication and publishing in a JWIF were not significantly associated either to individual researcher characteristics or with preferences for specialisation of the editorial strategy in HPSR or in the LA region.

## Discussion

The present article analysed the volume of papers, HPSR specialisation, editorial governance and quality (as judged by their IF) of research journals publishing papers pertaining to HPSR in LA countries. On this basis, a survey was undertaken with researchers publishing in these journals to identify preference regarding specific publication strategies – HPSR specialised versus more generic health or public health journals; open access versus subscription-based journals; focus on the LA regional versus more generic or global remit of the journal; English versus Spanish or Portuguese language, and the IF status of the journal.

This is the first study we are aware of that addresses the development of research journals as an area of concern in efforts to strengthen the HPSR system. To address this concern, we developed three indicators, namely the potential of an editorial policy, the feasibility of publishing in journals using an open access business model, and the challenges of publishing in new journals awaiting an IF. The three indicators have internal consistency, while only the indicator of ‘Potential’ – with the best consistency – was also the only one with significant associations to preferences for journal specialisation.

Limitations to this research are its focus on the sphere of journals indexed by Medline and, for some analyses, restriction to those with an IF. MeSH indexing has a delay of a few months and up to a year, depending on the journal’s impact factor [19]. This delay left out some titles from the study in the last year of analysis, affecting the relative count of LA HPSR papers vis-à-vis the global count if these papers are published with a higher frequency in low IF journals. Restricting papers to those indexed in Medline left out papers published in journals that are only indexed in other databases such as Lilacs. Our determination of a higher rate of growth for LA HPSR papers relative to papers for all regions of the world is, therefore, an underestimation. Restricting analysis to Medline-indexed papers did not alter our findings on the situation of LA HPSR publications published in journals with an IF, as it is safe to assume that they are all indexed in Medline.

The sampling framework for researchers was based on Medline indexing for a period of 7 years, with the possibility that emails had changed during the period, leading to unrecoverable addresses. Data for editorial governance was limited to the identification of membership within editorial boards and this, in turn, was limited to the identification of probable nationality based on Spanish or Portuguese surnames. Response to the survey in English or Spanish by Portuguese speaking researchers could have restricted access, although this is considered a minor limitation.

The LA region production of HPSR papers is growing twice as rapidly than the global production, although total papers are still three times below what would be expected given the region’s share of the global population. Production would be expected to be even greater than global averages given that most countries of the region are placed in the upper middle-income category. The greater growth of papers within a broad range of journals attests to a growing field with opportunity to leverage the region’s potential. Furthermore, global connectivity of HPSR researchers in upper middle-income countries as measured by co-authorship is almost on par with that in high-income countries [5]. This situation suggests the benefits that could accrue from a strategy of regional specialisation in a highly context-sensitive subject matter.

Educational policies as well as research capacity strengthening in countries of LA have led to increased publications, particularly in open access journals. In Brazil, one of the major drivers of the increase in publications in HPSR was the expansion of graduate programmes, with articles focused on the area of collective health quadrupling in number between 1996 and 2016 [20]. In Mexico, graduate programmes in collective health, public health and specifically in health systems research were markedly strengthened between the mid-70s and the mid-80s [21]. Research capacity strengthening policies across most countries in the region have privileged papers published in research journals with an IF as a major component of individual researcher performance evaluation. It must be stressed, however, that research authorities, as in the case of Mexico, value publications in new journals still lacking an IF as long as the journal demonstrates quality and rigorous peer review [22]. These policies have also led to increased funding for article processing charges for open access journals.

In this context, the low level of participation of editorial board members from the LA region is a concern, suggesting a limited capacity to promote and peer review research papers addressing regional and national priorities with optimal quality. The capacity to promote journals and to target research dissemination to policy-makers in the region may also be limited. However, evidence from research on the relationship between the IF of business journals and the national diversity of their board members suggests that diversity may not in itself be an important factor to ensure impact, as suggested by Petersen and Vogel [23]. Nonetheless, the national diversity of board members in journals that may be focusing on countries with similar income levels – as was the case with the business journals – may have a different influence on IF than diversity across countries with widely diverging health systems and income levels. This is an area that warrants further research.

While IF is not the only relevant nor even the best criterion to assess the quality of journals [24, 25], the low levels attained in this indicator by most mixed-topic journals published in the region does suggest an area of concern. This is the case particularly considering the attention being given by research authorities in the region to performance evaluation based on publications in journals with an IF. The researcher survey attested to a preference to publish in IF journals, something that has been suggested to lead to a systematic bias favouring international journals without staff or peer-review networks expert with health systems in the LA region, and therefore may not be the best qualified to promote the required evidence base [26].

The absence of journals specialised in HPSR and in the LA region is also a concern. It should be noted that not only Europe, the United States and Canada but also Africa have national or regional specialised journals in different fields of HPSR (for example, *African Journal of Health Professions Education*, *African Journal of Primary Health Care and Family Medicine*, *European Journal of Health Economics*, *Scandinavian Journal of Primary Health Care*), while most HPSR papers worldwide are published in specialised journals. Furthermore, HPSR papers for the region are mostly published in Spanish or Portuguese in public health journals. This situation suggests that the most qualified international researchers publishing in HPSR-specialised journals may not be prominent members of peer-review networks in LA journals.

The situation with journals publishing HPSR in the LA region warranted a survey to obtain researcher preferences regarding the potential of an editorial strategy that could be implemented through a new journal focusing on HPSR for the region, supported by a well-balanced Editorial Board comprised of internationally recognised researchers and policy-makers, published in English through an open access business model that could ensure the greatest dissemination.

Researchers for the most part viewed journal specialisation as a facilitator of quality publications, with specialisation in HPSR receiving greater support than specialisation on the LA region (86.5%). However, specialisation in the LA region has a stronger association with the proposed editorial policy, as expressed by the indicator of ‘Potential’. This suggests that an approach to strengthen the number and quality of HPSR publications in the LA region should consider both regional and HPSR specialisation.

The survey supported an editorial strategy based on open access, a commitment to publish within 3 months and with the support of a robust on-line submission platform. Also considered as important facilitators were a rigorous peer-review process supported by recognised regional health policy and systems researchers and the promotion of special issues on regional priorities. Researchers also considered as facilitators, although of less importance, an Editorial Board comprised in its majority of prominent LA researchers and policy-makers, and the publication by a well-renowned, international publisher.

The benefits of open access publications tend to outweigh the costs. However, upfront payment for open access is challenged by predominant opinion that it is difficult to earmark research funds to pay for open access, while only a minority of researchers declared that their institutions have agreements with open access publishers. It is interesting to note that residence of researchers in high-income countries outside LA (constituting 20% of the total, while including Chile still as an upper middle-income country) is not significantly associated to a perception of lower barriers to open access publication.

While researchers prefer to publish in journals with an IF, they also show a clear disposition to publish in a journal within a path to obtaining an IF in the context of special issues with prominent researchers and if the journal makes efforts to target dissemination to specific audiences. Publishing at no cost to the researcher or the institution during this period would facilitate the success of a new journal.

The survey suggests that English language publication with optional Spanish or Portuguese translations could be a strategy to facilitate quality HPSR publications in the LA region. There is no question that the use of English language for scientific publications is increasing, with at least 75% of periodical articles in the social sciences and the humanities at global level being written in English [27]. Furthermore, up to 94.5% of social science journals indexed by Thomson SSCI are published in English, and only 0.48% in Spanish and Portuguese [28]. Publishing in languages other than English is losing attraction among authors for whom English is not their mother tongue and that wish to be at the top of their scientific communities [27]. Furthermore, publishing and reading science in a non-native language has been found to reduce emotive biases when considering difficult moral choices, as is common in HPSR [29–31]. Science writing in Spanish and Portuguese will remain strong in the LA region, not only for the communication of research results at the national level but also for communication across the Spanish and Lusophone speaking countries. In this context, publications in the English language strengthen scientific multilingualism in the region [32, 33].

Scientific writing in English is also controversial as it places non-native English speakers at a disadvantage and risks imposing language-based biases to the presentation of research problems and results [34]. Writing science in English among Spanish speaking Mexican scientists was shown to increase difficulty by 23%, dissatisfaction by 11% and anxiety by 21% in comparison to writing in Spanish [35]. The cost of scientific writing in English can be mitigated through strengthening individual competence in ‘academic English’, facilitating the use of literacy brokers and training reviewers to avoid standardising native English styles and cultural biases as well as stigmatising the translation into academic English of diverse cultural standards [28].

## Conclusions

HPSR publications for the LA region are lagging behind those for high-income countries with respect to quality and specialisation, while peer review and dissemination may be restricted due to publication mostly in Portuguese or Spanish or by journals without sufficient expertise in the region. Researchers publishing on HPSR in LA are supportive of editorial strategies enabling a growing specialisation of publications in the HPSR field and particularly in the LA region. This support can be interpreted as a felt need among researchers towards greater specialisation in the field and towards seeking greater global recognition for their research. Researchers also support an open access publication characterised by a recognised Editorial Board, quality peer review, timely processing and a robust platform.

The preferences expressed by HPSR researchers in the LA region could be fulfilled by existing journals if they increased their expertise and their focus on HPSR and on the region through special sections or other editorial strategies. However, the support for specialisation both in HPSR and in the LA region, for academic English as the main language of communication and for an expert Editorial Board, all point towards the support for the launching of a new journal. The survey also indicates a new open access journal is feasible, while the challenges faced by a new journal in gaining an IF can be overcome through specific strategies. Global experience with academic English as a medium of scientific communication also points towards benefits and strategies to overcome its costs.

This research suggests the merits of assessing new publications in HPSR based on the potential of an editorial policy, the feasibility of open access publication and of the challenges faced by the waiting period prior to obtaining an Impact Factor. To ensure success, a new journal should clearly differentiate itself in terms of HPSR for the LA region and should strengthen the community of health policy and systems researchers to focus on literacy and peer-review skills. Special issues should be promoted through a priority-setting process to attract prominent authors, develop the audience and attain an IF.

Economic barriers to open access need not be a deterrent as efforts can be made to secure funding for special issues. Such funding could come from regional and national, large-scale research projects as well as from government ministries of health or science and technology institutes, from regional multi-lateral agencies such as the Inter-American Development Bank, and from non-profit donors. Funding should be sufficient to cover article processing charges as well as an executive editorial team, capacity strengthening and dissemination efforts.

Health systems in the region have developed since the 1930s following models that were not always best suited to addressing local realities. Reforms in the 80s and 90s led to a wide array of health systems with important lessons for equity, quality and efficiency. Academic institutions in the region are well developed and ready to assume greater responsibility for strengthening their capacity to publish HPSR at the global level as well as to invite greater global participation in addressing regional research problems. A journal on HPSR informed by a regional perspective could contribute to further deepening the analysis of challenges, promoting research priorities and consolidating evidence-informed decision-making.

## Supplementary information

**Additional file 1.** Appendix 1. Academic institutions of respondents. Appendix 2. Respondents’ countries of residence.

## Data Availability

The data used for this publication is available from Dr. Miguel A. González Block.

## Abbreviations

HPSR: Health policy and systems research
IF: Impact Factor
JWIF: Journals without an Impact Factor
LA: Latin America(n)
