# Supplementary material for: Health policy and systems research publications in Latin America warrant the launching of a new specialised regional journal
Source: Health Res Policy Syst. 2020 Jun 5;18:59. doi: 10.1186/s12961-020-00565-1 (PMC7275341; doi:10.1186/s12961-020-00565-1)
Supplement: Supplementary file 1 — Additional file 1. Appendix 1. Academic institutions of respondents. Appendix 2. Respondents’ countries of residence. [file 12961_2020_565_MOESM1_ESM.zip › Additional file 1.docx]

# Appendix 1. Academic Institutions of respondents

| **Institution** | ***n*** | **Percentage** |
| --- | --- | --- |
| Escola Nacional de Saúde Pública/ Fiocruz | 18 | 6.4 |
| Instituto Nacional de Salud Pública | 10 | 3.5 |
| Universidad Veracruzana | 7 | 2.5 |
| Universidad de Buenos Aires | 6 | 2.1 |
| Universidad Federal do Rio Grande (UFRG) | 6 | 2.1 |
| Universidade do Estado do Rio de Janeiro (UERJ) | 6 | 2.1 |
| Universidade Federal do Pelotas | 6 | 2.1 |
| Universidad de Chile | 5 | 1.8 |
| Universidad Nacional Autónoma de México | 5 | 1.8 |
| Universidad Nacional de Colombia | 5 | 1.8 |
| Universidade Federal do Minas Gerais (UFMG) | 5 | 1.8 |
| Universidade Federal do Santa Catarina | 5 | 1.8 |
| Universidade do Brasília | 4 | 1.4 |
| Escola Paulista de Medicina - Universidade Federal de São Paulo | 4 | 1.4 |
| Universidade Federal do Bahía, Brasil | 4 | 1.4 |
| Universidad de Antioquia | 3 | 1.1 |
| Universidad de los Andes | 3 | 1.1 |
| Universidad Nacional de Costa Rica | 3 | 1.1 |
| Universidad Nacional de Rosario | 3 | 1.1 |
| Universidad Peruana Cayetano Heredia | 3 | 1.1 |
| Consejo Nacional de Investigaciones Científicas y Técnicas, Argentina | 2 | 0.7 |
| Brown University School of Public Health | 2 | 0.7 |
| Health Institute, São Paulo State Health Secretariat, Brazil | 2 | 0.7 |
| Institute of Tropical Medicine of Amberes, Belgium | 2 | 0.7 |
| Instituto Mexicano del Seguro Social | 2 | 0.7 |
| Instituto Nacional de Psiquiatría Ramón de la Fuente Muñiz | 2 | 0.7 |
| London School of Hygiene & Tropical Medicine | 2 | 0.7 |
| McGill University | 2 | 0.7 |
| Organización Panamericana de la Salud | 2 | 0.7 |
| Pontifia Universidade Católica do Rio Grande do Sul | 2 | 0.7 |
| Pontificia Universidad Javeriana, Colombia | 2 | 0.7 |
| The Johns Hopkins Bloomberg School of Public Health | 2 | 0.7 |
| Universidad Autónoma del Estado de Hidalgo | 2 | 0.7 |
| Universidad Científica del Sur, Perú | 2 | 0.7 |
| Universidad de San Carlos de Guatemala | 2 | 0.7 |
| Universidad del Desarrollo, Chile | 2 | 0.7 |
| Universidad ICESI, Colombia | 2 | 0.7 |
| Universidad Juárez del Estado de Durango | 2 | 0.7 |
| Universidad Norte de Parana | 2 | 0.7 |
| Universidade estadual de Ponta Grossa | 2 | 0.7 |
| Universidade Federal do Triângulo Mineiro | 2 | 0.7 |
| University of North Carolina at Chapel Hill | 2 | 0.7 |
| University of Texas at el Paso | 2 | 0.7 |
| University of Uppsala, Sweden | 2 | 0.7 |
| Aga Khan University, Pakistan | 1 | 0.4 |
| Antwerp Institute of Tropical Medicine | 1 | 0.4 |
| Centro de Estudios para la Equidad y Gobernanza en los Sistemas de Salud, Guatemala | 1 | 0.4 |
| Centro de Investigaciones Regionales, UADY | 1 | 0.4 |
| Centro de Investigaciones y Estudios Superiores en Antropología Social | 1 | 0.4 |
| Centro Universitario Mauricio de Nassau | 1 | 0.4 |
| Claustro Universitario de Chihuahua | 1 | 0.4 |
| Comisión Económica para América Latina CEPAL | 1 | 0.4 |
| Consejo Latinoamericano de Ciencias Sociales | 1 | 0.4 |
| Consejo Nacional de Investigaciones Científicas y Técnicas | 1 | 0.4 |
| Dartmouth College | 1 | 0.4 |
| Faculdade de Enfermagem | 1 | 0.4 |
| Faculdade de Medicina de Jundiaí | 1 | 0.4 |
| Faculdade de Medicina de São José do Rio Preto | 1 | 0.4 |
| Facultad Latinoamericana de Ciencias Sociales | 1 | 0.4 |
| Federal University of Para Brazil | 1 | 0.4 |
| Federal University of Sergipe | 1 | 0.4 |
| Federal Univesity of Ceará | 1 | 0.4 |
| Florida International University | 1 | 0.4 |
| Fundación Universitaria de Ciencias de la Salud | 1 | 0.4 |
| Fundación Universitaria del Área Andina | 1 | 0.4 |
| Hanoi University of Public Health | 1 | 0.4 |
| Harvard University | 1 | 0.4 |
| Health Policy Research unit at Consorci de Salut i Social de Catalunya | 1 | 0.4 |
| Hospital Docente Clínico Quirúrgico Joaquín Albarrán | 1 | 0.4 |
| Hospital Infantil de México Federico Gómez | 1 | 0.4 |
| Hospital Universitari Vall d'Hebron | 1 | 0.4 |
| Imperial College London | 1 | 0.4 |
| Indiana University | 1 | 0.4 |
| Institute for Health Metrics and Evaluation, University of Washington | 1 | 0.4 |
| Instituto de Efectividad Clínica y Sanitaria | 1 | 0.4 |
| Instituto de Nutrición de Centroamérica y Panamá | 1 | 0.4 |
| Instituto de Salud Carlos III | 1 | 0.4 |
| Instituto Federal de Educação Ciência e Tecnologia do Ceará | 1 | 0.4 |
| Instituto Nacional de Salud, Perú | 1 | 0.4 |
| Instituto Tecnológico de Santo Domingo de República Dominicana | 1 | 0.4 |
| International Institute for Population Sciences | 1 | 0.4 |
| King’s College London | 1 | 0.4 |
| McMaster University | 1 | 0.4 |
| Oregon Health & Science University | 1 | 0.4 |
| Pontificia Universidad Católica del Perú | 1 | 0.4 |
| Queens University | 1 | 0.4 |
| School of Public Health - University of São Paulo | 1 | 0.4 |
| Southern Institute of Science and Technology | 1 | 0.4 |
| State University of New York at Stony Brook | 1 | 0.4 |
| The George Washington University | 1 | 0.4 |
| Umeå University, Sweden | 1 | 0.4 |
| Universidad Anahuac | 1 | 0.4 |
| Universidad Andres Bello | 1 | 0.4 |
| Universidad Autónoma de Madrid | 1 | 0.4 |
| Universidad Autónoma de Nuevo León | 1 | 0.4 |
| Universidad Autónoma de San Luis Potosí | 1 | 0.4 |
| Universidad Autónoma del Estado de Morelos | 1 | 0.4 |
| Universidad Católica los Ángeles de Chimbote | 1 | 0.4 |
| Universidad CES Medellin | 1 | 0.4 |
| Universidad de Alberta | 1 | 0.4 |
| Universidad de Ciencias Médicas de la Habana | 1 | 0.4 |
| Universidad de Costa Rica | 1 | 0.4 |
| Universidad de El Salvador | 1 | 0.4 |
| Universidad de Guadalajara | 1 | 0.4 |
| Universidad de Holguin | 1 | 0.4 |
| Universidad de la Costa, Perú | 1 | 0.4 |
| Universidad de la Frontera | 1 | 0.4 |
| Universidad de la Sabana, Colombia | 1 | 0.4 |
| Universidad de Monterrey | 1 | 0.4 |
| Universidad de San Martín de Porres | 1 | 0.4 |
| Universidad de Tulane | 1 | 0.4 |
| Universidad del Bosque | 1 | 0.4 |
| Universidad Industrial de Santander | 1 | 0.4 |
| Universidad Juárez Autónoma de Tabasco | 1 | 0.4 |
| Universidad Libre de Colombia | 1 | 0.4 |
| Universidad Nacional Autónoma de Honduras | 1 | 0.4 |
| Universidad Nacional de la Patagonia | 1 | 0.4 |
| Universidad Nacional de la Plata | 1 | 0.4 |
| Universidad Nacional de Luján | 1 | 0.4 |
| Universidad Nacional del General Sarmiento | 1 | 0.4 |
| Universidad Nacional Mayor de San Marcos | 1 | 0.4 |
| Universidad Pablo de Olavide | 1 | 0.4 |
| Universidad popular de la Chontalpa | 1 | 0.4 |
| Universidad San Francisco de Quito | 1 | 0.4 |
| Universidade Autonoma do Lisboa | 1 | 0.4 |
| Universidade do Lisboa | 1 | 0.4 |
| Universidade do Vale do Rio Dos Sinos | 1 | 0.4 |
| Universidade Federal de Goias | 1 | 0.4 |
| Universidade Federal de Mato Grosso do Sul | 1 | 0.4 |
| Universidade Federal de São João del Rei, Divinópolis | 1 | 0.4 |
| Universidade Federal do Piauí | 1 | 0.4 |
| Universidade Publica do Campinas, Brazil | 1 | 0.4 |
| University Hospital Care Medical Centre | 1 | 0.4 |
| University Hospital, Germany | 1 | 0.4 |
| University of Arizona | 1 | 0.4 |
| University of Bergen | 1 | 0.4 |
| University of Denver | 1 | 0.4 |
| University of Massachusetts Medical School | 1 | 0.4 |
| University of Missouri | 1 | 0.4 |
| University of Nebraska-Lincoln | 1 | 0.4 |
| University of Ottawa | 1 | 0.4 |
| University of Oxford | 1 | 0.4 |
| University of Pittsburgh | 1 | 0.4 |
| University of Region of Joinville | 1 | 0.4 |
| University of South Florida | 1 | 0.4 |
| University of Texas Rio Grande Valley | 1 | 0.4 |
| University of Toronto | 1 | 0.4 |
| University of Uberaba | 1 | 0.4 |
| University of Warwick | 1 | 0.4 |
| Veterans’ health administration | 1 | 0.4 |
| Not identified | 17 | 6 |
| **Total** | **282** | **100** |
