# Supplementary material for: Health policy and systems research publications in Latin America warrant the launching of a new specialised regional journal
Source: Health Res Policy Syst. 2020 Jun 5;18:59. doi: 10.1186/s12961-020-00565-1 (PMC7275341; doi:10.1186/s12961-020-00565-1)
Supplement: Supplementary file 1 — Additional file 1. Appendix 1. Academic institutions of respondents. Appendix 2. Respondents’ countries of residence. [file 12961_2020_565_MOESM1_ESM.zip › Additional file 2.docx]

# Appendix 2. Respondents’ countries of residence

| **Country** | **n** | **Percentage** |
| --- | --- | --- |
| Brazil | 98 | 32.8 |
| Mexico | 42 | 14.0 |
| United States of America | 29 | 9.4 |
| Colombia | 28 | 9.4 |
| Argentina | 22 | 7.4 |
| Chile | 15 | 5.0 |
| Peru | 12 | 4.0 |
| Canada | 8 | 2.7 |
| Spain | 6 | 1.7 |
| Costa Rica | 5 | 1.7 |
| England | 5 | 1.7 |
| Guatemala | 4 | 1.3 |
| Cuba | 3 | 1.0 |
| Ethiopia | 2 | 0.7 |
| Honduras | 2 | 0.7 |
| Belgium | 2 | 0.3 |
| Francia | 2 | 0.7 |
| Sweden | 2 | 0.3 |
| Vietnam | 1 | 0.3 |
| Pakistan | 1 | 0.3 |
| India | 1 | 0.3 |
| China | 1 | 0.3 |
| Ecuador | 1 | 0.3 |
| El Salvador | 1 | 0.3 |
| Germany | 1 | 0.3 |
| Norway | 1 | 0.3 |
| Netherlands | 1 | 0.3 |
| Portugal | 1 | 0.3 |
| Dominican Republic | 1 | 0.3 |
